# Chorioamnionitis and Subsequent Lung Function in Preterm Infants

**DOI:** 10.1371/journal.pone.0081193

**Published:** 2013-12-05

**Authors:** Marcus H. Jones, Andréa L. Corso, Robert S. Tepper, Maria I. A. Edelweiss, Luciana Friedrich, Paulo M. C. Pitrez, Renato T. Stein

**Affiliations:** 1 Institute of Biomedical Research, and School of Medicine, Pontifícia Universidade Católica do Rio Grande do Sul, Porto Alegre, Brazil; 2 Department of Pediatrics, James Whitcomb Riley Hospital for Children, Herman B. Wells Center for Pediatric Research, Indiana University, Indianapolis, Indiana, United States of America; 3 Department of Pathology, School of Medicine and Hospital de Clínicas de Porto Alegre, Universidade Federal do Rio Grande do Sul, Brazil; 4 Neonatology Section, Hospital de Clínicas de Porto Alegre, Brazil; The Ohio State Unversity, United States of America

## Abstract

**Objective:**

To explore the relationship between prematurity, gender and chorioamnionitis as determinants of early life lung function in premature infants.

**Methods:**

Placenta and membranes were collected from preterm deliveries (<37 weeks gestational age) and evaluated for histological chorioamnionitis (HCA). Patients were followed and lung function was performed in the first year of life by Raised Volume-Rapid Thoracic Compression Technique.

**Results:**

Ninety-five infants (43 males) born prematurely (median gestational age 34.2 weeks) were recruited. HCA was detected in 66 (69%) of the placentas, and of these 55(58%) were scored HCA Grade 1, and 11(12%) HCA Grade 2. Infants exposed to HCA Grade 1 and Grade 2, when compared to those not exposed, presented significantly lower gestational ages, higher prevalence of RDS, clinical early-onset sepsis, and the use of supplemental oxygen more than 28 days. Infants exposed to HCA also had significantly lower maximal flows. There was a significant negative trend for z-scores of lung function in relation to levels of HCA; infants had lower maximal expiratory flows with increasing level of HCA. (*p* = 0.012 for FEF_50_, *p* = 0.014 for FEF_25–75_ and *p* = 0.32 for FEV_0.5_). Two-way ANOVA adjusted for length and gestational age indicated a significant interaction between sex and HCA in determining expiratory flows (p<0.01 for FEF_50_, FEF_25–75_ and p<0.05 for FEV_0.5_). Post-hoc comparisons revealed that female preterm infants exposed to HCA Grade 1 and Grade 2 had significant lower lung function than those not exposed, and this effect was not observed among males.

**Conclusions:**

Our findings show a sex-specific negative effect of prenatal inflammation on lung function of female preterm infants. This study confirms and expands knowledge upon the known association between chorioamnionitis and early life chronic lung disease.

## Introduction

Premature birth can lead to a wide range of early life consequences for the immature lung and most of these are likely associated with increased respiratory morbidity in the first years of life. Of all main factors related to poor lung development in premature infants, gestational age is the best predictor of respiratory outcomes, but sex also plays an important role. [1], [2] Male preterm infants have a higher incidence rate of hospitalizations, bronchiolitis, and recurrent wheeze in the first years of life. [3]. One previous study from our group has shown that male preterm infants present up to 20% lower flows, as measured during the first three months of life, when compared to girls, at corresponding two full weeks of gestation. [4] This ventilatory disadvantage explains, at least in part, the increased frequency of obstructive respiratory morbidity among male preterm infants.

Another aspect that may influence respiratory morbidity in the first years of life is the fact that infant girls, who are more likely to be born with the benefit of larger airways compared to boys, seem to lose this advantage if exposed to pre-natal maternal smoking [5], [6] or due to an inadequate breastfeeding scheme. [7], [8] Prenatal exposure to inflammation has also been generally considered relevant in the respiratory prognosis of preterm infants. [9] The concept of exposure of the fetal lung to infection and inflammation, promoting in one direction accelerated maturation, and simultaneously, in an opposite direction, development of chronic lung disease (CLD) of prematurity has been previously demonstrated by a number of studies. [10]–[14] Several of these have shown an increased incidence of CLD or bronchopulmonary dysplasia (BPD) in infants with a previous diagnosis of histological chorioamnionitis (neutrophilic infiltration of membranes, HCA), [15]–[17] or signs of systemic inflammatory responses. [18]–[20] In contrast, two large reports found no significant association between HCA and either CLD or BPD. [21], [22] One potential limitation of most studies may be the use of a cut-off parameter of oxygen at 36 weeks post-menstrual age (PMA) as a surrogate for CLD. It has been previously postulated that this binary outcome is not ideal to describe the continuous effects of prematurity and inflammation on lung structure and function. [23] Another limitation of previous studies is that inflammation has not been evaluated controlling for important risk factors such as sex and gestational age in a single explanatory model. Therefore, the contribution of chorioamnionitis on lung development, through early life lung function measurements, should be investigated in the context of these intervening variables.

In this study, we explore the relation between prematurity, sex and chorioamnionitis as determinants of lung function during the first year of life, assessed by objective measurements. We hypothesized that maximal expiratory flows are reduced in those preterm infants exposed to inflammation, and that the effect is modulated by sex and gestational age.

### Patients and Methods

The study was approved by the ethics committee of our institution (PUCRS, Brazil) and an informed written consent was obtained from all parents.

We conducted a prospective cohort study including all newborn premature infants (aged less than 37 weeks gestational age). All patients were recruited at birth in a single academic hospital in Porto Alegre, Brazil, for a period of 12 months.

Gestational age was determined by last menstrual period, and confirmed by early obstetric ultrasound (in the first 12 weeks of gestation) and clinical assessment by a neonatologist. [24] Infants with major congenital malformations, chromosomal syndromes, and confirmed HIV exposure were excluded from the study.

All NICU procedures and events were recorded prospectively by one of the investigators (ALC). A newborn was considered to have Respiratory Distress Syndrome (RDS) when there was requirement of FIO2≥0.40, presence of characteristic chest X-ray, and need of exogenous surfactant replacement. Small-for-gestational-age (SGA) was defined by birth weight below the 10^th^percentile for gestational age. [25] Clinical early-onset sepsis was diagnosed by the attending physician by clinical criteria. [26] The diagnosis of the Patent Ductus Arteriosus (PDA) was made by clinical signs and confirmed with an ultrasound exam. There were no interventions on clinical decisions or diagnoses by the study team, and these were subject to the attending physician. All clinical information was based on chart reviews.

### Histologic Chorioamnionitis

Five tissue samples were obtained from each placenta (including umbilical cord, membranes and chorionic plate), fixed with 10% buffered formalin, embedded in paraffin and tissue block and stained with hematoxylin and eosin. Histologic examination was performed by a pathologist (MIE) blinded to the clinical information and scored as “no chorioamnionitis”, “Grade 1” (Redline [27] maternal Stage 1 or 2 and Grade 1) and “Grade 2” (Redline [27] maternal Stage 3 and Grade 2). Signs of fetal inflammatory response, characterized by chorionic vasculitis were also recorded.

### Lung Function Tests

Lung function testing was assessed by the Raised Volume-Rapid Thoracic Compression Technique (RV-RTC), after sedation with chloral hydrate (50–80 mg/kg). [28] Lung function tests were performed after 40 weeks of postconceptional age, in the first year of life. Infants were weighed, measured, and set in supine position, with an inflatable jacket wrapped around their abdomen and chest. Pulse oximeter monitoring was used during the tests. A facemask was positioned over the infant’s face, covering mouth and nose, and the cervical region was maintained in an over-extended position.

Lung inflation pressure was set at 30 cm H_2_O and, at this point, thoracic compression was initiated and maintained until residual volume was reached. Forced expiratory maneuvers were repeated with increases of 5 to 10 cm H_2_O in jacket pressure until maximum expiratory flows were obtained. The best curve was selected as that with the highest product of Forced Vital Capacity (FVC) and Forced Expiratory Flow Between 25% and 75% of FVC (FEF_25–75_) [29].

### Statistical Analysis

Statistical analysis of the data was performed using the statistical program SPSS, version 17 (SPSS, Inc., Chicago, IL). Quantitative and qualitative variables were described, respectively, through means/SD, median/range and frequencies/percentiles. Group characteristics (male *vs*. female, HCA *vs*. no HCA) were compared by Mann-Whitney test, and Pearson Chi-square. Z-scores of the lung function variables were used, as a length-adjustment approach. [30] The associations of the three levels of HCA with qualitative and continuous variables were assessed by Pearson Chi-square and Jonckheere–Terpstra trend test, respectively.

Stepwise multiple regression analysis was used to determine the independent relations of the perinatal variables to lung function. Perinatal factors were: sex, HCA, tobacco smoking exposure during pregnancy, gestational age, birth weight (BW), SGA, clinical early-onset sepsis, PDA, maternal corticosteroid use, prolonged premature rupture of membranes (>18 h).

The data were analyzed by two-way ANOVA (sex by HCA) and ANCOVA, including length and gestational age as covariates. For post-hoc analysis, the Holm-Sidak test was used. For all statistical analyses, p-values below 0.05 were considered significant.

## Results

95 premature infants were recruited (43 males) at the time of birth. Median (range) gestational age was 34.2 (24 to 36.8) weeks and median birth weight was 2,255 (710 to 3,550) grams. Fifty-five (58.3%) of the subjects were white. RDS was observed in 15 (16%) infants. While 26 (27%) had a diagnosis of clinical early-onset sepsis, blood culture was positive in only two subjects. Supplemental oxygen for more than 28 days was required by 10 (10.5%) of these infants. HCA was detected in 66 (69%) of the placentas, and of these 55(58%) were scored Grade 1, and 11(12%) Grade 2. Fetal inflammatory response was detected in only 6 infants, two with chorionic vasculitis, three with umbilical vasculitis and one with necrotizing funisitis.

As shown in Table 1, birth weight and gestational age were not significantly different between males and females, while prevalence of PDA, mechanical ventilation and use of supplemental oxygen for more than 28 days being significantly higher among male infants. Infants exposed to HCA Grade 1 and Grade 2, when compared to those not exposed, presented significantly lower gestational ages, higher prevalence of RDS and clinical early-onset sepsis, and the use of supplemental oxygen more than 28 days.

**Table 1:** Subjects characteristics, stratified by sex and chorioamnionitis.

	Sex		Histologic Chorioamnionitis			
*Perinatal Data*	Male(n = 43)	Female(n = 52)	None(n = 29)	Grade 1(n = 55)	Grade 2(n = 11)	Grade 1 and 2(n = 66)
Gestational Age (w)	34.0 (24–36.8)	34.6 (26–36.8)	35 (31.3–36.8)	34.7 (26–36.8)	31.7 (24–32.7)^##^	34.2 (24–36.8)*
Birth weight (g)	2145 (710–3550)	2345 (745–3495)	2445 (1335–3100)	2330 (710–3550)	1510 (715–2560)#	2208 (710–3550)
Smoking exposurepregnancy	8 (18.6%)	10 (19.2%)	7 (24.1%)	7 (12.7%)	4 (35.4%)	11 (16.7%)
Antenatal steroids	19 (44.2%)	18 (34.6%)	11 (37.9%)	20 (36.4%)	6 (54.5%)	26 (39.4%)
PROM>18 h	4 (9.1%)	7 (13.5%)	3 (10.3%)	6 (10.9%)	2 (18.2%)	8 (11.9%)
SGA	1 (2.3%)	7 (13.5%)	3 (10.3%)	5 (9.1%)	0 (0.0%)	5 (7.6%)
RDS	11 (25.6%)	4 (7.7%)*	0 (0.0%)	12 (21.8%)	3 (27.3%)^##^	15 (22.7%)**
SupplementalOxygen >28 days	9 (20.9%)	1 (1.9%)**	0 (0.0%)	7 (12.7%)	3 (27.3%)#	10 (15.2%)*
Clinical early-onsetsepsis	12 (27.9%)	14 (26.9%)	2 (6.9%)	19 (34.5%)	5 (45.5%)^##^	24 (36.4%)**
PDA	8 (18.6%)	2 (3.8%)*	0 (0.0%)	8 (14.5%)	2 (18.2%)	10 (15.2%)*
Mechanical Ventilation	11 (25.6%)	4 (7.7%)*	2 (6.9%)	9 (16.4%)	4 (36.4%)	13 (19.7%)

PROM (Premature Rupture of Membranes >18 h), SGA (Small-for-gestational-age), RDS (Respiratory Distress Syndrome), PDA (Patent Ductus Arteriosus).

Values expressed as number (%) or median(range).

* p<0.05; **p<0.01; for Mann-Whitney Test for continuous variables and for Pearson Chi-square for qualitative variables between male versus female preterm infants and between None versus HCA Grade 1 and HCA Grade 2 combined.

# p<0.05; ^##^p<0.01; for Jonckheere–Terpstra trend test for continuous variables and for Pearson Chi-square for qualitative variables between None, HCA Grade 1 and HCA Grade 2.

**Figure 1:**
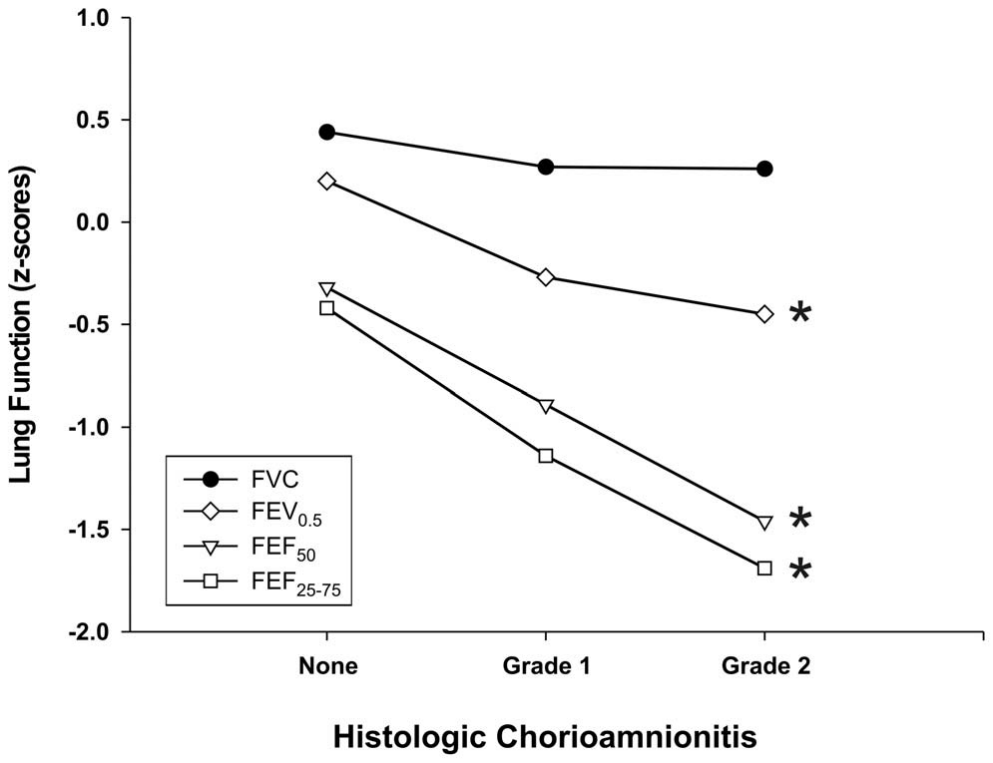
Lung function variables, expressed as mean z-score by HCA level in premature infants. *p<0.05 for Jonckheere–Terpstra trend test.

In Table 2, the results of lung function testing, expressed as z-scores, show that males had significantly lower expiratory flows, while FVC values were not significantly different between both sexes. The analysis of lung function by HCA level show significantly lower expiratory flows when HCA Grade 1 and Grade 2 were combined and compared to No HCA. In addition, the Jonckheere-Terpstra test revealed a significant negative trend for z-scores of lung function in relation to the levels of HCA; infants had lower maximal expiratory flows with increasing level of HCA. (*p* = 0.012 for FEF_50_, *p* = 0.014 for FEF_25–75_ and *p* = 0.32 for FEV_0.5_)(Figure 1).

**Table 2:** Lung function values, stratified by sex and chorioamnionitis.

	Sex		Histologic Chorioamnionitis			
Lung Function Test	Male(n = 43)	Female(n = 52)	None (n = 29)	Grade 1 (n = 55)	Grade 2 (n = 11)	Grade 1 and 2 (n = 66)
Age (corrected, weeks)	22.1±9.5	21.8±10.2	25,5±10,81	18,9±8,43	27,5±8,72#	21.1±10.8*
Weight (kg)	6.4±1.9	6.2±1.4	7,2±1,72	5,8±1,39	6,4±1,68#	6.1±1.7*
Weight/age	−0.25±1.63	0.18±1.03	0,43±1,131	−0,12±1,27	−0,64±1,92	−0.17±1.42
Length (cm)	61.4±5.83	61.6±5.30	64,1±5,19	59,9±5,37	62,7±4,45#	61±6*
Length/age	−0.38±1.40	0.37±1.13*	0,26±1,04	−0,01±1,40	−0,36±1,49	−0.05±1.41
FVC	0.33±1.01	0.31±0.85	0,44±0,99	0,27±0,93	0,26±0,69	0.24±0.90
FEF_50_	−1.57±1.65	−0.14±1.24**	−0,32±1,83	−0,89±1,38	−1,46±1,80#	−0.98±1.45*
FEF_25–75_	−1.82±1.81	−0.29±1.43**	−0,42±1,88	−1,14±1,64	−1,69±1,93#	−1.23±1.68*
FEV_0.5_	−0.53±1.20	0.17±0.94*	0,20±1,36	−0,27±1,00	−0,45±0,79#	−0.31±0.96*
FEV_0.5_/FVC	−1.62±1.44	−0.27±1.17**	−0,50±1,54	−1,00±1,33	−1,33±1,80#	−1.03±1.41*

Lung function, Weight/age, and Length/age expressed in Z scores. Data are mean±SD.

* p<0.05; **p<0.01; for Mann-Whitney Test for continuous variables between male versus female preterm infants and between None versus HCA Grade 1 and HCA Grade 2 combined.

# p<0.05 for Jonckheere–Terpstra trend test for continuous variables between None, HCA Grade 1 and HCA Grade 2.

There was a significant negative correlation between HCA and Gestational age in both sexes (r = −0.302, *p* = 0.049 for males and r = −0.336, *p* = 0.015 for females). HCA level was also significantly correlated to lower maximal flows in female preterm infants (r = −0.315, p = 0.023 for FEF_25–75_ and r = −0.307, p = 0.027 for FEF_50_) but not in males.

### Multivariate Analysis

The differences between sexes in lung function values remained significant after adjusting for length, weight (expressed as Z score), and gestational age in a multivariate model. Female preterm infants had, on average, expiratory flows 119 mL/s (31%) higher than males for FEF_50_. For FEF_75_, FEF_25–75_ and FEV_0.5_ the difference was 76 mL/s (49%), 109 mL/s (34%) and 20 mL (12%), respectively. FVC values were not significantly different for male and female subjects. In this multivariate analysis, adjusting for sex, length, and gestational age, there was no significant effect on lung function of HCA, race, smoking exposure during pregnancy, PPROM, RDS, antenatal steroids, supplemental oxygen for more than 28 days and SGA on measured lung function variables.

**Figure 2:**
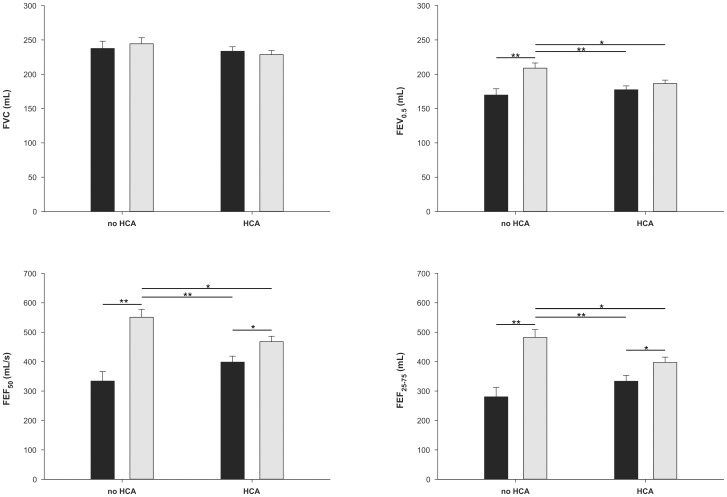
Lung function adjusted for body length and gestational age in male (black) and female (gray) premature infants. Data are represented as the mean (+SD). Sixty-six (35 female) were exposed to HCA (combined Grade 1 and Grade 2) and 29 not exposed to HCA (17 female). There was a significant sex by HCA interaction for FEF_50_ (F = 8.76; p = 0.004), FEF_25–75_ (F = 8.11; p = 0.005) and FEV_0.5_ (F = 4.81; p = 0.031). Post hoc analyses revealed a significant reduction in lung function in exposed female preterm infants when compared to females not exposed to HCA. The effect of exposure to HCA was not significant in males. *p<0.05, **p<0.01 (Post hoc Holm-Sidak test).

Two-way ANOVA adjusted for length and gestational age indicated a significant interaction between sex and HCA in determining expiratory flows (p<0.01 for FEF_50_ and FEF_25–75_ and p<0.05 for FEV_0.5_). The interaction between sex and HCA was significant with both HCA categorized in three levels (i.e., None, Grade 1, and Grade 2) or two levels (None, and Grade1 plus Grade2). Post-hoc comparisons by Holm-Sidak test revealed that female preterm infants exposed to HCA Grade 1 and Grade 2 had significant lower lung function than those not exposed, and this effect was not observed among males. Unexposed female preterm infants had significantly higher maximal expiratory flows when compared to males and exposed females. The results are presented in Figure 2.

## Discussion

Our main finding is that maternal chorioamnionitis, as diagnosed by independent histological evaluation, is associated with lower lung function in female premature infants, and this effect is not observed in males. The magnitude of the effect of chorioamnionitis in maximal flows was not minimal. We observed a reduction between 15% and 20% in FEF_50_, FEF_75_, and FEF_25–75_ values in females exposed to intrauterine inflammation. This finding offers additional insight on the role of inflammation in the development of chronic lung disease of prematurity and may improve our understanding on the variability of lung function in preterm infants. The reasons for this selective negative effect on females, and why males would be protected from inflammation are yet unknown.

Being male or female has an important impact on lung development and susceptibility to respiratory diseases. [31] In this sense, when compared to girls, boys have a clear disadvantage in the early postnatal period [32] and during the first years of life [3]. Our results suggest a “masculinization effect” of HCA on lung function of female preterm infants. Our data, adjusted by length and gestational age, shows that girls unexposed to HCA have expiratory flows 71% higher than unexposed boys in FEF_25–75_, and this is reduced to only 18% among those exposed to HCA. (Figure 2) Chorioamnionitis seems to act by narrowing this gender-driven respiratory developmental gap in preterm infants.

Similar to our main finding of lower lung function among baby girls exposed to HCA, exposure to maternal smoking during pregnancy has been associated with lower lung function, an effect that seems more clearly detected also in female infants. [5], [6] Tager and colleagues describe that female infants exposed to smoke *in utero* had lower flows (VmaxFVC) compared to those not exposed, but this was not observed in boys. In the same line of thought, female infants who were adequately breastfed were protected against severe bronchiolitis (a quite likely surrogate for lower lung function) in contrast with those who were not [7], [8], and this was also especially relevant for female preterm infants [33]. We speculate, considering the reported sex specific respiratory effects of these “insults” (i.e., chorioamnionitis, smoke exposure and lack of breastfeeding), that the ventilatory advantage of female infants is dependent on optimal conditions, either during pregnancy or in the early postnatal period.

Previous studies point to an association between prematurity and chorioamnionitis and obstructive respiratory illnesses in children. [34], [35] Our data confirm these previous findings and advances in showing a sex specific effect not previously evaluated in a more complex model. However, our results conflict with a recent study in preterm infants that showed no significant effect of chorioamnionitis on lung function. [36] These authors measured FRC, respiratory resistance, and compliance in infants born below 32 weeks of gestational age. Differences in subjects and methodology may explain the observed discrepancy. Respiratory resistance, the main outcome measured in that study is affected by upper airway size and patency, which can be explained by suboptimal measurements in patients with severe obstruction. In this British study, the population consisted of very low birth weight (VLBW) infants. [36] It is important to take into account that extreme prematurity may have an overwhelming effect when included in a model, to the point that variables such as concomitant chorioamnionitis may not be statistically detected as a significant risk factor. One way to see our data in a broader perspective is that, for the overall population, gestational age is a much stronger explanatory variable than is chorioamnionitis for lung function. The observation that female infants are specially affected by HCA does not overrule the previous statement.

We found a large number of subjects exposed to HCA in our sample (65%). This could be a result of lower social conditions, poorer public health care, or even race, as previously reported [22], [37]. Alternatively, our sample may reflect a selection bias for the more severe cases, since subjects were recruited in a large tertiary hospital with a referral NICU for high-risk pregnancies. In addition, the definition criteria for inflammation used in this study, of five or more polymorphonuclear leukocytes per high-powered field may be considered a “low threshold”, and this fact could increase the diagnosis of HCA. It has been demonstrated that HCA diagnosis (or its prevalence level in a given population) is strongly influenced by the definition adopted and by exclusion criteria and ethnicity. [38] With similar criteria to the one used in defining HCA in our study (i.e., 5 neutrophils per field), Holzman et al. analyzing placenta from full term infants reported HCA in 54–76% among European descents and in 64–85% African-Americans. [38] What seems important here is that the discussion of the validity of these cut-off values for defining HCA are not central to our findings, since they are helpful in discriminating a group of female premature infants at risk for lower expiratory flows. This data on HCA may be potentially useful clinical information for physicians who need to evaluate risk of respiratory morbidity early in life.

One limitation of this study is the lack of a satisfactory marker for duration and intensity of the inflammatory exposure. The intensity of neutrophilic infiltration at the time of delivery cannot be used to estimate the extension or severity of inflammation during the whole pregnancy period. The histological findings obtained at birth are poor descriptors of the intrauterine milieu and lung fetal exposure throughout the gestation period. [10] Another weakness of the study is that we have a single lung function measurement made in a wide range of ages, from 4 to 52 weeks of corrected age. This allows for an increased variability due to additional adverse events, such as lower respiratory infections that may occur after birth and before lung function testing. Still, this limitation would also favor the null hypothesis, as it would tend to dilute the effects of the prenatal and early postnatal events. Nevertheless, the result of exposure to intrauterine infection and/or inflammation upon airway function remained significant among female infants.

In summary, our study presents original data on the association between HCA and lung function measured in the first year of life, assessed by the RV-RTC technique in preterm infants. We have found a reduction in maximal expiratory flows in female preterm infants exposed to HCA, an effect not observed in males. These results imply that there may be a selective negative effect of inflammation on the development of the lung, especially among female preterm infants.
